# COVID-19 impact on the psychological health of Latinx transgender and non-binary individuals in mainland United States and Puerto Rico: A mixed-methods study

**DOI:** 10.21203/rs.3.rs-1498987/v1

**Published:** 2022-04-13

**Authors:** Alixida Ramos-Pibernus, Sheilla Rodríguez-Madera, Ernesto Rosario-Hernández, Fabián Moreta-Ávila, Julián Silva-Reteguis, Eliut Rivera-Segarra

**Affiliations:** Ponce Health Sciences University; Florida International University; Ponce Health Sciences University; Independent Researcher; Independent Researcher; Ponce Health Sciences University

**Keywords:** Latinx trans and non-binary, COVID-19 pandemic, Puerto Rico, Trans health

## Abstract

**Background:**

The COVID-19 pandemic continues to generate an unprecedented impact on all aspects of everyday life across the world. However, those with historically and currently marginalized identities (i.e., gender or ethnicity) who already experience a wide range of structural inequities have been disproportionally impacted. LTNB are a particularly at-risk population as they lie at the intersection of race/ethnicity, gender identity, language, migration status, geographical location, among others, which could further increase their COVID-19 and other health-related risks and disparities. The objective of this study was to examine the impact of key social determinants of health (i.e., gender identity, country, health insurance, employment) among a sample of LTNB individuals.

**Methods:**

The team implemented a cross-sectional exploratory design with an online survey technique using the secure web platforms REDcap and SurveyMonkey. A total of 133 participants completed the online survey. Most of the sample self-identified as transwomen (38.8%), transmen (26.3%), and non-binary (21.8%) between the ages of 21 to 72. All participants were Latinx living in either Puerto Rico (47.7%) or mainland United States (52.3%). Descriptive statistics, reliability tests, Mann-Whitney and rapid thematic analysis test were conducted.

**Results:**

Findings show that most participants were always (38.1%) or almost always (33.3%) worried about contracting COVID-19. Individuals living in Puerto Rico reported more difficulties than those residing in the mainland US regarding COVID-19 impact on psychosocial, emotional, and COVID-related thinking. Most participants’ answers for the COVID-19 open-ended questions focused on three main domains: income, access to trans-affirmative health care, and coping strategies.

**Discussion:**

Findings evidence that although most of LTNB participants were negatively impacted by the COVID-19 pandemic in multiple aspects of their lives, those living in Puerto Rico experienced these differently when compared to those in mainland US. More research is needed to understand better the mechanisms and pathways through which this context specifically impacts LTNB health and wellbeing, particularly in Puerto Rico. This study could help shape the public health response taking into account the geographical location and other intersectional identities that play critical roles in the production and reproduction of inequities.

## Introduction

The COVID-19 pandemic continues to generate an unprecedented impact on all aspects of everyday life across the world. However, those with historically and currently marginalized identities (i.e., gender or ethnicity) who already experience a wide range of structural inequities have been disproportionally impacted (1). Such is the case of transgender and non-binary individuals (TNB). Some significant challenges for TNB, particularly during the early stages of the COVID-19 pandemic, were the reduced access to gender-affirming care (2–4) and the delay in gender-affirming surgeries (5, 6), which were considered to be non-essential medical care as a result of the pandemic. This leads to increased psychosocial challenges and mental health problems such as persistent gender dysphoria (7).

Further evidence shows the particularly detrimental psychosocial and emotional consequences of the COVID-19 pandemic for TNB, including increased unemployment and lack of secure housing (8), increased anxiety and depression (9), unmet mental health needs (10), increased use of alcohol (11), and higher prevalence of suicide thoughts (12, 13). Some of these disparities can be explained by the social determinants of health influencing the health status of minoritized TNB individuals (8, 14). Nevertheless, little is known about their impact among TNB with multiple marginalized intersecting identities, such as Latinx trans and non-binary individuals (LTNB).

LTNB are a particularly at-risk population as they lie at the intersection of race/ethnicity, gender identity, language, migration status, geographical location, among others, which could further increase their COVID-19 and other health-related risks and disparities (2, 15, 16). For example, several studies have consistently found Latinxs to experience higher COVID-19 disease burden, transmission, positivity rates, and mortality than non-Latinxs (17–21). However, there is still a scarcity of empirical studies examining the psychosocial impact of the pandemic, particularly among LTGNB individuals. The few available studies have only focused on transwomen (22) and sexual minority men (11); have relied on reduced samples of Latinx individuals (8, 23), or are non-empirical commentaries (24). This study fills this gap by focusing on a sample of LTNB individuals from mainland United States and Puerto Rico. The objective of this study was to examine the impact of key social determinants of health (i.e., gender identity, country, health insurance, employment) among a sample of LTNB individuals.

## Methods

### Procedures

This research is part of a larger study that examines barriers and facilitators for cancer screening among LTNB individuals. After obtaining Institutional Review Board (IRB) approval, the team implemented a cross-sectional exploratory design with an online survey technique using the secure web platforms REDcap and SurveyMonkey. Recruitment was conducted by availability between July 2020 and April 2021, during the first wave of the pandemic. The inclusion criteria were: (1) self-identify as trans, non-binary, or any other self-identifying term used to represent gender diversity; (2) 21 years of age (the age of adulthood in Puerto Rico) or older; and (3) Identify as Latinx. With the collaboration of key community researchers, members of the research team distributed the survey on social media platforms (Facebook, Instagram, Twitter), sent the link via email and text message, and distributed flyers with QR codes in LGBT centers and clinics. Participants received an Amazon card for $25 as an incentive for completing the survey.

### Participants

A total of 133 participants completed the online survey. Most of the sample self-identified as transwomen (38.8%), transmen (26.3%), and non-binary (21.8%) between the ages of 21 to 72. In terms of sexual orientation 33.2% identified as heterosexual, followed by pansexual with 17.3%. All participants were Latinx living in either Puerto Rico (47.7%) or mainland United States (52.3%). Most of them were single (59.4%) at the time of the study, with a monthly income of less than USD 1,500. Table 1 presents a more detailed description of the sample.

### Measures

For the purpose of this manuscript, we present data regarding participants’ COVID-19 experience. As part of our larger study, the investigators designed and administered an online survey which collected information about participants’: 1) sociodemographic information; 2) COVID-related worries (1 item Likert scale ranging from “1” never to “5” always); and 3) impact of the COVID-19 pandemic on their transition/affirmation process (1 checklist item), and 4) social, emotional, and physical impact of the COVID-19 pandemic (comprised of 4 checklist items). Additionally, we included two open-ended questions to examine participants’: 1) general experience regarding the COVID-19 pandemic and 2) specific COVID-19 pandemic-related coping strategies.

### Data Analysis

Descriptive statistics (mean, frequencies, and normality) and reliability tests were conducted. Considering the homogeneity of the sample and the low percentage of missing values (< 0.4%), single imputation (mean substitution) was used to replace missing values with the mean for the entire series (25). Scores deviated from a normality distribution. Therefore, the analyst proceeded to sum all selected checklist options per item (social, emotional, physical, covid related thinking and transition) in order to conduct a Mann-Whitney test to compare COVID-19 impact by country.

To examine the open-ended questions’ qualitative data, the analyst implemented a rapid thematic analysis, a new innovative analytic technique that allowed to obtain targeted qualitative data in a shorter period (26, 27). Responses were divided into three main areas related to 1) income and 2) access to gender-affirming care, and 3) coping strategies.

## Results

Findings show that most participants were always (38.1%) or almost always (33.3%) worried about contracting COVID-19. In terms of social impact, participants reported loss of employment (31.6%) and loss of social support network (34.6%). More than half of the participants reported feeling stressed (76.7%), anxious (69.2%), and sad (63.3%). Half of them (50.4%) mentioned avoiding thinking about what might happen to prevent stress. More than half of the participants (54.9%) recognized that their diet was affected. Finally, in terms of their gender affirmation/transition process, 36.4% admitted they had had difficulties with hormonal treatment follow-up. Detailed results can be found in Table 2.

Additionally, as seen in Table 3, results show that individuals living in Puerto Rico reported more difficulties than those residing in the mainland US regarding COVID-19 impact on psychosocial, emotional, and COVID-related thinking. In terms of their effect sizes, psychosocial and COVID-related thinking impact have effect sizes considered to be small, although psychosocial was close to a medium effect size. However, emotional impact has a medium effect size (28).

### Qualitative data

Most participants’ answers for the COVID-19 open-ended questions focused on three main domains: income, access to trans-affirmative health care, and coping strategies. Table 4 presents sample quotes from each of the three identified themes.

## Discussion

Findings evidence that although most of LTNB participants were negatively impacted by the COVID-19 pandemic in multiple aspects of their lives, those living in Puerto Rico experienced these differently when compared to those in mainland US. Similar to other studies, mental health challenges were identified, including increased worry about contracting COVID-19 (5, 8). Moreover, feelings of stress, anxiety, sadness, and hopelessness were prevalent among the sample regarding the current pandemic. These findings show how the COVID-19 pandemic seem to be an additional stressor linked to mental health challenges.

In terms of social-related challenges, the loss of social support network was a key finding as a high percentage of participants identified this as a salient difficulty. Previous research has evidenced that having a social support network is linked to community resilience/resistance(29). Thus, lacking adequate social support networks could directly impact their wellbeing. An additional important finding was that almost a third of the sample lost their employment due to the pandemic. This is crucial as financial needs among this population have been extensively documented as a fundamental social determinant of health linked to the manifestation of oppression due to their gender and racial/ethnic identities and with detrimental consequences to their health and wellbeing (30, 31). Taken together, quantitative and qualitative findings suggest that the already precarious economic situation of LTNB seemed to be exacerbated by COVID-19.

Another area of concern was related to their trans-affirmative health care. Similar to other studies, participants identified difficulties related to their gender-affirming care, specifically delays in surgical procedures and follow-up of their hormonal treatment (2, 32). Qualitative results also suggest that some participants postponed their hormone therapy, and others mentioned their difficulties getting medical appointments or buying medication refills. This is particularly worrisome as reduced access to gender-affirming care has been linked to increasing mental health vulnerability, including increased suicide behaviors (7). For example, Flaherty et al. (6) have argued for the need to prioritize gender-affirming care as urgent or time-sensitive during the COVID-19 pandemic. We support and extend the argument by highlighting the need to urgently and specifically consider LTNB individuals based on this study’s findings.

Additionally, one of the main findings of this study is that participants living in Puerto Rico during the COVID-19 pandemic seemed to be experiencing more psychosocial and emotional challenges than those living in the mainland US. One potential explanation for this is the experience of constant stressors associated with the unstable social and political landscape in Puerto Rico during the past decade. For example, since 2008, Puerto Rico has experienced an economic recession, bankruptcy, austerity measures imposition, the ousting of a governor, the impact of two major hurricanes (Irma and María), and a swarm of earthquakes in 2020, all before the pandemic unfolded. This has led some researchers to posit that the main threat to Puerto Ricans living in the archipelago during the COVID-19 pandemic is precisely the context mentioned above (33–35). These study findings suggest more research is needed to understand better the mechanisms and pathways through which this context specifically impacts LTNB health and wellbeing, particularly in Puerto Rico. Moreover, to eventually highlight the areas and systems that need to be put in place to create more sustainable & holistic support in benefit of these communities, during and post-pandemic times.

Finally, besides the identified challenges, findings also evidence specific strategies used by participants to resist and address the COVID-19 related challenges. For example, some of them engaged in entertainment activities, disconnected from the news, or engaged in alternative supporting networks and activities such as Reiki and religion. Recognizing the strengths and specific strategies used by LTNB individuals during challenging events such as the COVID-19 pandemic is key to tailoring targeted interventions, disaster preparedness, and fostering their health and wellbeing.

This study has several limitations. First, the survey was only conducted during the first wave of the COVID-19 pandemic. Thus, no follow-up information about the impact of the pandemic was gathered at a different point in time. Secondly, the study did not include standardized instruments to assess the mental health symptoms of participants as this was not part of the aims of the broader study. Data reported here regarding their mental health and social challenges were self-reported. Finally, the sampling strategy used was by availability which limits the representative and generalizability of the findings.

Despite the limitations, this study highlights some of the challenges and experiences of LTNB individuals who are heavily neglected in the scientific literature. This study could help shape the public health response taking into account the geographical location and other intersectional identities that play critical roles in the production and reproduction of inequities.

## Figures and Tables

**Table 1:** Sociodemographic characteristics of participants

Demographic category	Freq. (%)
Assigned sex at birth	
Female	68 (51.1%)
Male	60 (45.1%)
Prefer not to answer	5 (3.8%)
Gender Identity*	
Woman	13 (9.8%)
Man	11 (8.3%)
Transwoman	45 (38.8%)
Transman	35 (26.3%)
Non-binary	29 (21.8%)
Sexual Orientation	
Heterosexual	43 (32.3%)
Homosexual	12 (9%)
Lesbian	8 (6%)
Bisexual	20 (15%)
Pansexual	23 (17.3%)
Other	18 (13.5%)
Prefer not to answer	9 (6.8%)
Country of residence	
Puerto Rico	63 (47.7%)
United States	69 (52.3%)
Marital Status	79 (59.4%
Single	
Legally married	15 (11.3%)
Living together	30 (22.6%)
Divorced or separated	4 (3%)
Prefer not to answer	5 (3.8%)
Education	
Did not complete high school	12 (9%)
High School	9 (6.8%)
Some college	34 (25.6%)
Associate degree	23 (17.3%)
Bachelor’s degree	37 (27.8%)
Graduate degree	12 (9%)
Prefer not to answer	6 (4.5%)
Employment (Check all that apply)	
Full-time job	53 (39.8%)
Part-time job	18 (13.5%)
Multiple jobs	14 (10.5%)
Unemployed	19 (14.3%)
Disability	2 (1.5%)
Monthly income	
$0-$100	28 (21.1%)
S101-$500	15 (12%)
$501-$1,000	19 (14.3%)
$1,101-$1,500	18 (13.5%)
$1,501-$2,000	17 (12.8%)
More than $2,000	23 (17.3%)
I don’t know or prefer not to answer	12 (9.1%)

Note. n = 133.

* As described by participants

**Table 2:** Frequency of Covid-19 experiences

Questions	Freq.	Percent
How frequently do you worried about contracting Covid-19		
Always	48	38.1%
Almost always	42	33.3%
Sometimes	26	20.6%
Almost never	9	7.1%
Never	1	.8%
*Social Impact*		
I have lost my employment	42	31.6%
I have lost my housing	12	9%
I have lost my social support network	46	34.6%
It has not impacted me	22	16.5%
*Emotional Impact*		
I have felt anxiety	92	69.2%
I have felt stress	102	76.7%
I have felt fear	78	58.6%
I have felt sadness	84	63.3%
I have felt hopelessness	72	54.1%
I have had changes in my sleeping patterns	72	54.1%
I have had changes in my appetite	51	38.3%
It has not affected me	8	6%
*Covid Related Thinking*		
I have nightmares or constant covid related thoughts	31	23.3%
I avoid thinking what might happen to avoid stress	67	50.4%
I am in a constant state of alert	57	42.9%
I have felt numbed or distant from what happens around me	30	22.6%
I have felt irritable	51	38.3%
*Physical Health Impact*		
I have lost my medical appointments	46	34.6%
I have not been able to do exercise	61	45.9%
My diet has been affected	73	54.9%
I have not been impacted	12	9%
*Transition Impact*		
I had to postpone a surgical procedure	24	18%
I have had difficulties with hormonal treatment follow-up	48	36.4%
It has not impacted me	43	32.2%

Note. n = 133.

**Table 3:** Comparison of impact indices LTNB individuals by country (Puerto Rico/United States) via Mann-Whitney tests

Impact Index	PR (n = 62)	US (n = 69)	Mann-Whitney’s U	Z-value	p-value	Effect Size (r)
	Mean Rank	Mean Rank				
Psychosocial	75.85	57.15	1528*	−2.825*	.005	.25
Social	66.99	65.11	2077	−0.347	.728	.03
Emotional	78.80	54.50	1345*	−3.707*	< .001	.32
Covid Related Thinking	73.37	59.38	1682*	−2.195*	.028	.19
Physical	71.22	61.31	1815	−1.575	.115	.14
Transition	60.65	70.80	1807	−1.875	.061	.16

Note. n = 131

* p < .05.

**Table 4:** Rapid qualitative analysis sample quotes

Theme	Definition	Sample quotes
Income worries	Participants’ descriptions about income challenges and worries due to the covid 19 pandemic.	1. I’m spending all my savings 2. I have faced financial limitations because I am the breadwinner in my family, and it has been complicated to manage all the expenses … 3. I would like the government to support us, the people who have lost their jobs and cannot generate income to pay the rent and other debts
Trans-affirmative health care	Participants’ descriptions about difficulties and worries for accessing gender-affirming care.	1. I had to postpone my initial appointment for hormone therapy 2. I have not been able to leave my home, so I have not seen a doctor or started the procedures 3. I missed my appointments, the medical orders expired, and wasn’t able to get new ones from my doctor
Coping strategies	Participant’s description of the ways in which they defy the challenges of COVID-19	1. online social support groups, Reiki and religion 2. finding forms of distraction such as reading, watching movies, and virtual shows… 3. detach myself from the news a bit
